# Interictal epileptiform activity in the acute stroke phase: an independent predictor of poor outcome

**DOI:** 10.1093/esj/aakaf001

**Published:** 2026-01-01

**Authors:** Giovanni Furlanis, Katerina Iscra, Edoardo Ricci, Michele Malesani, Gabriele Prandin, Emanuele Vincis, Laura Mancinelli, Federica Palacino, Magda Quagliotto, Paola Caruso, Marcello Naccarato, Miloš Ajčević, Paolo Manganotti

**Affiliations:** Clinical Unit of Neurology, Department of Medicine, Surgery and Health Sciences, University Hospital and Health Services of Trieste, University of Trieste, Trieste, Italy; Department of Engineering and Architecture, University of Trieste, Trieste, Italy; Clinical Unit of Neurology, Department of Medicine, Surgery and Health Sciences, University Hospital and Health Services of Trieste, University of Trieste, Trieste, Italy; Clinical Unit of Neurology, Department of Medicine, Surgery and Health Sciences, University Hospital and Health Services of Trieste, University of Trieste, Trieste, Italy; Clinical Unit of Neurology, Department of Medicine, Surgery and Health Sciences, University Hospital and Health Services of Trieste, University of Trieste, Trieste, Italy; Clinical Unit of Neurology, Department of Medicine, Surgery and Health Sciences, University Hospital and Health Services of Trieste, University of Trieste, Trieste, Italy; Clinical Unit of Neurology, Department of Medicine, Surgery and Health Sciences, University Hospital and Health Services of Trieste, University of Trieste, Trieste, Italy; Clinical Unit of Neurology, Department of Medicine, Surgery and Health Sciences, University Hospital and Health Services of Trieste, University of Trieste, Trieste, Italy; Clinical Unit of Neurology, Department of Medicine, Surgery and Health Sciences, University Hospital and Health Services of Trieste, University of Trieste, Trieste, Italy; Clinical Unit of Neurology, Department of Medicine, Surgery and Health Sciences, University Hospital and Health Services of Trieste, University of Trieste, Trieste, Italy; Clinical Unit of Neurology, Department of Medicine, Surgery and Health Sciences, University Hospital and Health Services of Trieste, University of Trieste, Trieste, Italy; Department of Engineering and Architecture, University of Trieste, Trieste, Italy; Clinical Unit of Neurology, Department of Medicine, Surgery and Health Sciences, University Hospital and Health Services of Trieste, University of Trieste, Trieste, Italy

**Keywords:** acute stroke, EEG, interictal epileptiform discharges, outcome prediction

## Abstract

**Introduction:**

Electroencephalography (EEG) features are emerging as valuable prognostic indicators in acute stroke. However, data on the predictive value of interictal epileptiform discharges (IEDs) remain limited. This study aimed to assess the prognostic role of IEDs in predicting functional outcomes in stroke patients without symptomatic seizures who underwent point-of-care EEG within 72 h of admission.

**Patients and methods:**

We retrospectively analysed the clinical, neurophysiological and neuroimaging data of acute stroke patients who underwent point-of-care EEG within 72 h of admission. Interictal epileptiform discharges were identified according to the International Federation of Clinical Neurophysiology criteria. A multivariate logistic regression model identified variables associated with modified Rankin scale (mRS) scores of 3-6 at 3 months.

**Results:**

Among 593 stroke patients (median age 77 years, range 22-98; median National Institutes of Health Stroke Scale [NIHSS] 5, range 0-25), 18.2% exhibited IEDs on EEG within 72 h of admission. At 3-month follow-up, 223 patients (37.6%) demonstrated poor functional outcome (mRS 3-6). The presence of IEDs on EEG (odds ratio [OR] = 1.088, *P* = .037), along with age (OR = 1.004, *P* < .001), NIHSS at admission (OR = 1.032, *P* < .001), premorbid disability (OR = 1.111, *P* < .001), hemorrhagic stroke (OR = 1.120, *P* < .001) and lesion extent (OR = 1.070, *P* < .001), was an independent predictor of poor clinical outcomes at 3 months (mRS 3-6). The logistic regression model, including these factors, achieved 81% accuracy in predicting functional outcomes.

**Conclusion:**

Early IEDs on EEG within 72 h are independent predictors of poor clinical outcomes (mRS 3-6) at 3 months. These findings underscore the importance of EEG monitoring in the acute phase of stroke and suggest that IED detection may serve as an additional prognostic marker.

## Graphical Abstract

**Graphical abstract:**
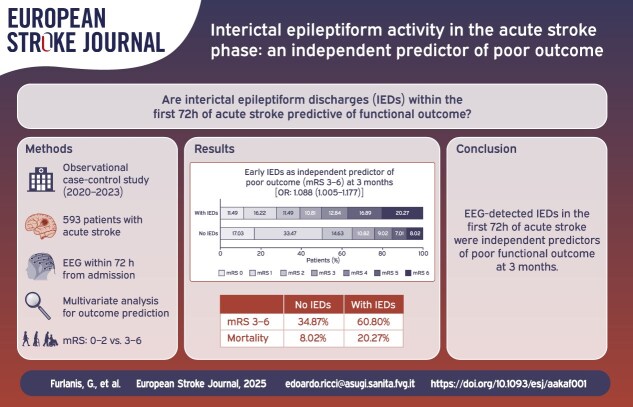

## Introduction

Acute stroke is a neurological emergency characterised by a decrease in regional cerebral blood perfusion that induces a reduction in oxygen and glucose.^1^ These sudden perfusion abnormalities cause alterations in the brain’s electrical activity^2^^,^^3^ as a manifestation of neurovascular coupling.^4^ Electroencephalography (EEG) is a non-invasive technique with high temporal resolution characterised by good sensitivity to acute changes in neural metabolism.^5^ However, EEG remains under-exploited in routine clinical practice for acute cerebrovascular disease. In the context of acute stroke, this technique is mainly applied to support the differential diagnosis between ischemic stroke and seizure stroke mimic, particularly in cases with unclear symptoms such as isolated aphasia.^6^

Prediction of clinical and functional outcome in the acute stroke patient is complex since it is determined by different clinical and radiological features.^7–10^ In recent years, some studies have been conducted to identify potential prognostic factors and improve models that can predict clinical outcomes and create personalised therapeutic interventions to improve patient care outcomes.^11–13^ Moreover, recent researches support the hypothesis that neurophysiological biomarkers, such as EEG, can provide valuable short- and long-term prognostic information for stroke patients.^14–19^ Therefore, the EEG in the acute phase can be useful for early prediction of the outcome and to allow better management of the patient in terms of therapy and rehabilitation. Quantitative features extracted from power spectral analysis of EEG tracings showed potential for outcome prediction. Indeed, EEG indices such as delta–alpha ratio and relative alpha power have been shown to correlate with National Institutes of Health Stroke Scale (NIHSS) scores at discharge (7 days)^14^ and at 30 days after stroke,^19^ as well as alpha relative power and the delta–theta–alpha–beta ratio were reported as significant predictors of post-stroke outcomes at both discharge^14^^,^^15^ and 12 months post-stroke.^15^ Moreover, acute symptomatic seizures were found to be independent predictors of mortality and remote symptomatic seizures predictors of functional outcome in the first year after stroke.^18^

Besides EEG slowing abnormalities, there is growing evidence of the presence of epileptiform patterns in patients with acute ischemic stroke. Recent studies have explored the prevalence of interictal epileptiform abnormalities (IEDs) and seizures during the acute or subacute phases of stroke.^20^^,^^21^ The prevalence of acute symptomatic seizures in patients with cerebrovascular events ranges between 4% and 10% and it is considered an unfavourable prognostic factor for functional outcomes.^22–26^ Interictal epileptiform abnormalities were observed in 25.7% of cases.^20^ Lasek-Bal et al. observed EEG epileptiform abnormalities in 44.27% of stroke patients; the most common findings were “Generalised Rhythmic Delta Activity (GRDA)” and sporadic discharges, predominantly localised to the hemisphere affected by the cerebrovascular event. However, there are still limited data regarding the presence of interictal epileptiform discharges (IEDs) during the acute phase of stroke and their predictive power in stroke patients, in particular in patients without clinical or electric seizures or those with a history of epilepsy or treatment with anti-seizure drugs.

Therefore, the aim of our study was to evaluate the possible prognostic role in terms of modified Rankin scale (mRS) at 3 months of the presence of IEDs in acute stroke patients without symptomatic seizures undergoing a point-of-care EEG in the first 72 h.

## Patients and methods

### Study population

We retrospectively analysed the clinical, neurophysiological and neuroimaging data of a cohort of consecutive acute stroke patients who were admitted to the Stroke Unit of the Neurology Unit of the Trieste University Hospital (Trieste, Italy) between September 2020 and May 2023 and who underwent an EEG within 72 h from admission as our standard internal protocol. All patients with ischemic or hemorrhagic stroke admitted to our Stroke Unit were included in the study without age limits or gender restrictions. Patients admitted to the Stroke Unit who were later determined to have a stroke mimic were excluded from the study. Additionally, patients with a history of epilepsy, those receiving antiepileptic drugs and those who experienced seizures or status epilepticus during hospitalisation were excluded. Patients with confirmed strokes who, for logistical or technical reasons, did not undergo an EEG were also excluded from the study. All patients with a suspected acute cerebrovascular event were assessed with a non-enhanced computed tomography (NECT) on admission, while only a proportion of them had a CT scan of the extra- and intracranial vessels and a CT perfusion. All patients without a confirmed lesion on the control CT scan underwent an MRI scan. All CT imaging (NECT, CTA and CTP) was performed with a 256-slice CT scanner (Brilliance iCT; Philips Medical Systems, Best, the Netherlands), while all MRI was performed with a 1.5 T or 3.0 T MRI scanner (MR 5300 1.5 T, Philips Medical Systems scanner or MR Ingenia Elition 3.0 T, Philips Medical Systems scanner). Two expert neurologists (M.N. and G.F.) analysed the neuroimaging data.

The following data of included patients were collected: (1) demographic details (age, sex); (2) comorbidities and acute conditions (atrial fibrillation [AF], diabetes, history of hypertension, smoke, dyslipidemia, chronic heart failure, coronaropathy, chronic kidney disease, diagnosis of cognitive impairment, diagnosis of previous stroke, acute infectious disease); (3) stroke severity and outcome (NIHSS ^27^ at admission, NIHSS at discharge, pre-stroke mRS,^28^ intrahospital mortality, mRS at discharge); (4) type of stroke (ischemic stroke, hemorrhagic stroke); (5) TOAST classification for ischemic stroke^29^; (6) lesion characteristics (lobar/lobar and basal ganglia/basal ganglia, supratentorial/infratentorial), side (right, left and bilateral), location of cerebral infarct area (frontal, parietal, temporal, insular, occipital, basal ganglia/thalamus, brainstem) and sum of lobes with an infarct area on NECT; (7) type of reperfusion treatment for ischemic stroke (thrombolysis, direct thrombectomy, thrombolysis plus thrombectomy); (8) hemorrhagic transformation for ischemic stroke (asymptomatic haemorrhage, symptomatic haemorrhage according to the ECAS II classification).^30^

Patients included in the study were therefore divided into 2 different groups: the group of patients with mRS at 3 months 0-2 (good outcome) and the group of patients with mRS at 3 months 3-6 (poor outcome). This study has been conducted according to the principles of the Declaration of Helsinki and was approved by the University of Trieste’s Ethics Committee.

### E‌EG acquisition and analysis

19 channel (10-20 system) 20-min standard clinical surface EEG was acquired within 72 h from admission by using the Be Plus PRO amplifier (EB NEURO, Florence, Italy) and Ag/AgCl electrodes. Electroencephalography signals were filtered by a second-order band-pass Butterworth filter with 0.1-30 Hz cut-off frequencies. Electroencephalography tracings were assessed by qualitative visual inspection, performed by expert neurologists (G.F. and P.M.) blinded to clinical and outcome data, in accordance with international criteria.^31^^,^^32^ All doubts were decided by consensus with another neurologist (P.C.). The inter-rater agreement for the classification of IEDs was calculated using Cohen’s κ coefficient (κ = 0.83). The EEG protocol included resting-state recordings with eyes closed and activation tests with eyes open, tailored to the patient’s clinical presentation. The IEDs were identified according to the International Federation of Clinical Neurophysiology (ie, IEDs were defined as di- or tri-phasic waves with sharp or spiky morphology distinct to background activity, typically followed by an associated slow after wave). Focal slowing was not considered as IED.^32^ The EEG tracings were classified as: (1) EEG without IEDs; (2) presence of IEDs; severity of IEDs^33^; presence of asymmetric IEDs to the side of the lesion. The frequency of IEDs was classified according to Kane’s glossary^31^ as follows: 1/10 s—abundant, 1/min but <1/10 s—frequent, 1/h but <1/min—occasional and <1/h—rare.

### Clinical assessment and outcome measures

All patients received standardised clinical and diagnostic assessment, during admission and after discharge. During admission and on discharge, it was measured the clinical outcome in terms of NIHSS^26^ (NIHSS at discharge). The NIHSS, consisting of 11 items to assess the main neurological functions, is the most adopted tool in the actual medical practice to evaluate stroke-related neurologic impairment at admission and evaluate clinical evolution and outcome. Two neurologists (E.V. and E.R.) conducted a phone interview or a medical examination 3 months after stroke to determine outcome. Functional outcome was measured by mRS, an ordinal scale with 7 categories ranging from 0 (no symptoms) to 6 (death): 0—no symptoms at all; 1—no significant disability despite symptoms, able to carry out all usual duties and activities; 2—slight disability, unable to carry out all previous activities, but able to look after own affairs without assistance; 3—moderate disability, requiring some help, but able to walk without assistance; 4—moderately severe disability, unable to walk and attend to bodily needs without assistance; 5—severe disability, bedridden, incontinent and requiring constant nursing care and attention; 6—dead. Good outcome class was defined with 3-month mRS ≤ 2, while poor outcome class was defined with 3-month mRS > 3.

### Statistical analysis

We performed all statistical analyses using Matlab (MathWorks Inc., Natick, MA). Descriptive statistics were presented as medians and interquartile ranges (IQRs) for continuous variables, and as counts and percentages (*n*, %) for categorical variables. Normality of distributions was examined using the Kolmogorov–Smirnov test. Comparative analyses between patients with good versus poor stroke outcomes were performed using Student’s *t*-test for continuous variables with normal distribution, or the Mann–Whitney *U* test for non-normally distributed data. Categorical variables were compared employing the chi-square test. Univariate and multivariate logistic regression analyses were also applied to a subset of selected features (sex, age, stroke risk factors, previous stroke, acute infection disease, NIHSS at admission, pre-stroke mRS, type of stroke, lesion characteristics and presence of IEDs) to identify predictors of outcome. In particular, variables associated with *P* values < .05 at univariate analysis were selected as candidate factors for the linear model. Odds ratios (ORs) with corresponding 95% confidence intervals (CIs) were calculated for the logistic regression model. Statistical significance was assumed at *P* < .05. Finally, the performance of the developed logistic regression model was evaluated based on accuracy and area under the curve (AUC) values.

## Results

**Figure 1:**
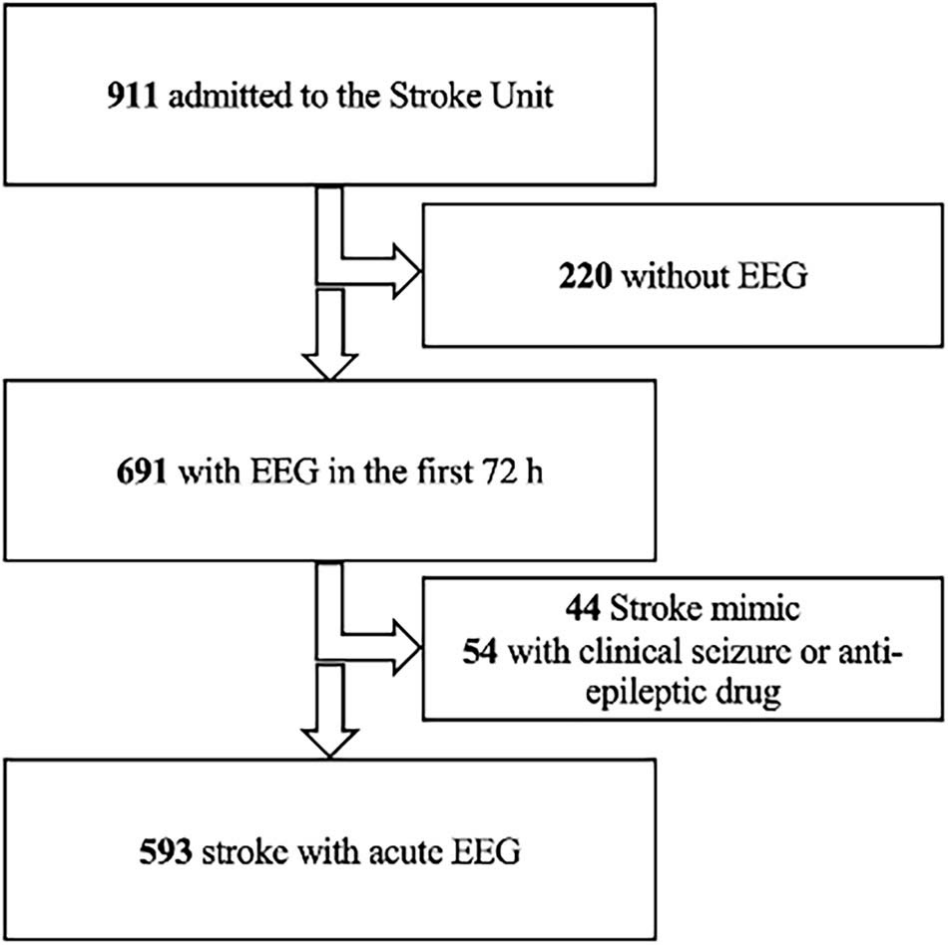
Study flow diagram.

From September 2020 to May 2023, 911 patients were admitted to the Stroke Unit, 691 of whom underwent EEG assessment during the subacute phase. Forty-four patients with stroke mimics and 54 patients who developed clinical seizures and received anti-epileptic therapy were excluded from the study. The final sample consisted of 593 stroke patients (median: 77 [22-98] year, 278 females) who underwent EEG within the first 72 h. The study flow diagram is presented as Figure 1.

Clinical and demographic data, comorbidities, lesion localization by neuroimaging (NECT) and stroke type are reported in Table 1. The majority of patients experienced ischemic strokes (516, 87%), while 77 (13%) had hemorrhagic strokes. The most common comorbidities among patients were hypertension (451, 76.1%), dyslipidemia (234, 63.2%) and atrial fibrillation (182, 30.7%). The median NIHSS score at admission was 5 (0-25). The cerebral infarct areas primarily involved the basal ganglia/thalamus (32.2%), followed by the parietal (29.5%), frontal (26.8%), temporal (22.3%), insular (15.9%) and occipital (14.3%) regions, with brainstem involvement in 5.7% of cases. Among patients with ischemic stroke classified according to the TOAST criteria, the majority had a stroke of undetermined cause (SUC, 33.3%), followed by those classified as cardioembolic (26.0%) and those with small artery occlusion (22.1%). Additionally, 12.6% had an atherothrombotic stroke, and 6.2% had a stroke due to other determined causes. In the ischemic stroke patient group, 32.2% received thrombolysis, 3.0% underwent direct thrombectomy and 10.5% received both thrombolysis and thrombectomy; asymptomatic haemorrhages occurred in 6.0% of patients, while symptomatic haemorrhages were observed in 9.1% of ischemic stroke patients.

**Table 1:** Demographic and clinical characteristics of the study cohort. Comparison of demographics and clinical characteristics between patients with good outcomes at 3 months (mRS 0-2) and those with poor outcomes at 3 months (mRS 3-6).

		mRS 0-2	mRS 3-6	*P*-value
	***n* = 593**	***n* = 370**	***n* = 223**	
**Demography**				
Age (years) [median (IQR)]	77 (67-93)	74 (64-81)	82 (74-86)	**<.001**
Females [*n* (%)]	278 (46.9)	159 (43)	76 (34.2)	**.014**
**Comorbidities and acute conditions**				
Atrial fibrillation [*n* (%)]	182 (30.7)	90 (24.3)	92 (41.3)	**<.001**
Diabetes mellitus [*n* (%)]	141 (23.8)	80 (21.6)	61 (27.4)	.112
Hypertension [*n* (%)	451 (76.1)	274 (74.1)	177 (79.4)	.141
Smoke [*n* (%)]	106 (17.9)	68 (18.0)	38 (17)	.680
Dyslipidemia [*n* (%)]	234 (63.2)	234 (63.2)	128 (57.4)	.157
Chronic heart failure [*n* (%)]	64 (10.8)	28 (7.6)	36 (16.1)	**.001**
Coronaropathy [*n* (%)]	83 (14.0)	44 (11. 9)	39 (17.5)	.057
Chronic kidney disease [*n* (%)]	82 (13.8)	46 (12.4)	36 (16.1)	.204
Cognitive impairment [*n* (%)]	61 (10.3)	26 (7.0)	35 (15.7)	**<.001**
Previous stroke [*n* (%)]	67 (11.3)	33 (8.9)	34 (15.3)	**.018**
Acute infectious disease [*n* (%)]	79 (13.3)	28 (7.6)	51 (22.9)	**<.001**
Body mass index (BMI) [median (IQR)]	26.6 (25-28)	26.7 (25-28.3)	26.5 (24.9-27.8)	.086
**Stroke severity and outcome**				
NIHSS at admission [median (IQR)]	5 (2-10)	4 (2-6)	11 (6-19)	**<.001**
NIHSS at discharge [median (IQR)]	1 (0-4)	0 (0-1)	7 (3-15)	**<.001**
Pre-stroke mRS [median (IQR)]	0 (0-1)	0 (0-0)	0 (0-2)	**<.001**
Intrahospital mortality [*n* (%)]	21 (3.5)	–	–	**–**
mRS at discharge [median (IQR)]	2 (1-5)	1 (1-2)	5 (4-5)	**<.001**
**Type of stroke**				
Ischemic [*n* (%)]	516 (87)	336 (90.8)	180 (80.7)	**<.001**
Hemorrhagic [*n* (%)]	77 (13)	34 (9.2)	43 (19.3)	**<.001**
**Neuroimaging**				
Lesion				
Lobar [*n* (%)]	298 (50.3)	167 (45.1)	131 (58.7)	**.001**
Lobar and basal ganglia [*n* (%)]	76 (12.8)	12 (3.2)	64 (28.7)	**<.001**
Basal ganglia [*n* (%)]	220 (37.1)	103 (27.4)	117 (52.5)	**<.001**
Supratentorial [*n* (%)]	527 (88.9)	324 (87.6)	203 (91.0)	.193
Infratentorial [*n* (%)]	66 (11.1)	46 (12.4)	20 (9.0)	.194
Side				
Right [*n* (%)]	202 (34.1)	117 (31.6)	85 (38.1)	.106
Left [*n* (%)]	264 (44.5)	160 (43.2)	104 (46.6)	.474
Bilateral [*n* (%)]	7 (1.2)	4 (1.2)	3 (1.4)	.773
Sum of lobes [median (IQR)]	1 (0-6)	1 (0-5)	2 (0-6)	**<.001**

Data are presented as means, medians (IQR) and frequencies. Significant *P*-values are highlighted in bold.

Abbreviations: IQR = interquartile range; mRS = modified Rankin scale; NIHSS = National Institutes of Health Stroke Scale.

The IEDs were observed in 108 patients (18.2%). In particular, 54 patients (9.1%) exhibited rare IEDs on the acute EEG, 49 patients (8.3%) had occasional IEDs, 4 patients (0.7%) had frequent IEDs and 1 patient (0.2%) showed abundant IEDs. Of 108 patients with interictal epileptiform abnormalities, 77 (71.3%) presented asymmetric IEDs. Furthermore, IEDs were observed in 91 of 516 patients with ischemic stroke (17.6%) and in 17 of 77 patients with hemorrhagic stroke (22.1%), with no statistically significant difference between the 2 groups (*P* = .43).

Table 1 also reports the comparisons of demographics and clinical features between patients with good outcomes at 3 months (mRS 0-2) and those with poor outcomes at 3 months (mRS 3-6). The mRS 0-2 patients were younger than mRS 3-6 ones (74 years vs 82 years; *P* < .001), female gender was more represented in the mRS 0-2 group (43.0% vs 34.2%; *P* = .014). Concerning the risk factor, atrial fibrillation (24.3% vs 41.3%; *P* < .001), chronic heart failure (7.6% vs 16.1%; *P* = .001), previous cognitive impairment (7.0% vs 15.7%; *P* < .001) and a previous stroke (8.9% vs 15.3%; *P* = .018) were more presented in the mRS 3-6 group. Patients with hemorrhagic stroke were more frequently observed in the mRS 3-6 group compared to the mRS 0-2 group (43 vs 34; *P* < .001). Concerning the neuroimaging, the median of the sum of lobes with pathological involvement was higher in the mRS 3-6 group (1 vs 2; *P* < .001). The significantly higher prevalence of IED was detected in patients with poor outcome mRS 3-6 compared to those with good outcome mRS 0-2 (26.5% vs 13.2%, *P* < .001).

**Figure 2:**
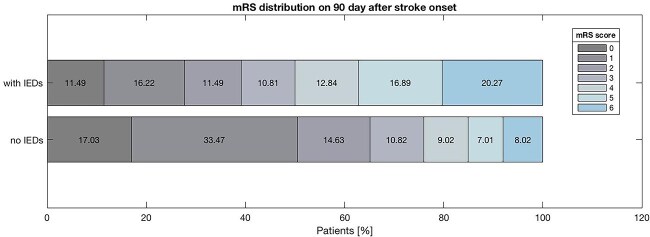
mRS distribution on 90 days after stroke onset.

The mRS distribution on 90 days after stroke onset of patients with epileptiform abnormalities in EEG and patients without it are depicted in Figure 2. It can be observed that the patients with EEGs showing IEDs exhibit a lower percentage of mRS scores between 0 and 2 compared to those without IEDs (39.20% vs 65.13%, *P* < .001). Furthermore, a greater percentage of mortality was detected in patients with EEGs displaying IEDs (20.27% vs 8.02%, *P* < .001).

A total of 57 patients (9.6%) underwent EEG within 24 h of symptom onset, while 536 patients (90.4%) underwent EEG between 24 and 72 h. Among those who received EEG within 24 h, 35 patients (59.6%) had a favourable outcome at 3 months (mRS 0-2), whereas 23 patients (40.4%) had a poor outcome (mRS 3-6). In the group that underwent EEG between 24 and 72 h, 335 patients (62.5%) had a favourable outcome and 201 (37.5%) had a poor outcome at 3 months.

**Figure 3:**
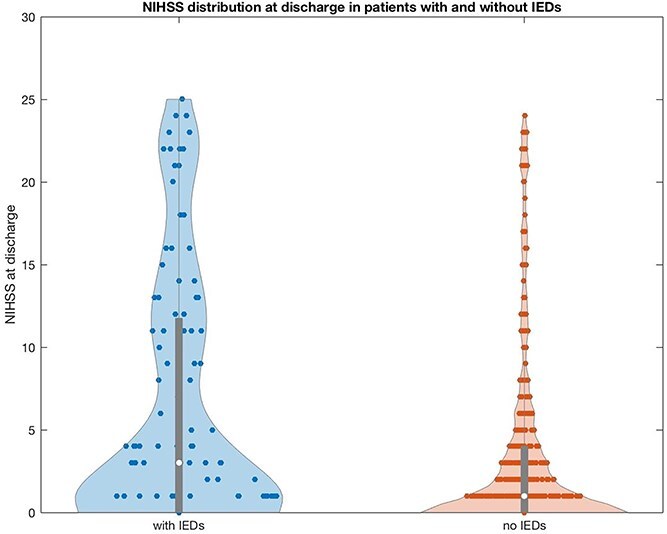
Violin plot showing the distribution of NIHSS scores at discharge in patients with and without IEDs. Abbreviation: IEDs = interictal epileptiform discharges.

Figure 3 depicts the violin plots of NIHSS distribution at discharge in patients with and without IEDs. The distribution in the IEDs group showed greater variability and a higher proportion of severe neurological deficits, whereas patients without IEDs had a more concentrated distribution with lower NIHSS scores, suggesting better functional outcomes.

Univariate regression analysis identified gender (OR = 1.103, *P* = .014), age (OR = 1.010, *P* < .001), cognitive impairment (OR = 1.247, *P* < .001), AF (OR = 1.205, *P* < .001), chronic heart failure (OR = 1.232, *P* = .001), previous stroke (OR = 1.160, *P* = .018), acute infectious disease (OR = 1.365, *P* < .001), NIHSS at admission (OR = 1.043, *P* < .001), pre-stroke mRS (OR = 1.170, *P* < .001), ischemic stroke (OR = 1.233, *P* < .001), lobar lesion (OR = 1.136, *P* = .001), sum of lobes (OR = 1.123, *P* < .001) and IEDs (OR = 1.231, *P* < .001) as significant parameters associated with patient outcomes.

**Figure 4:**
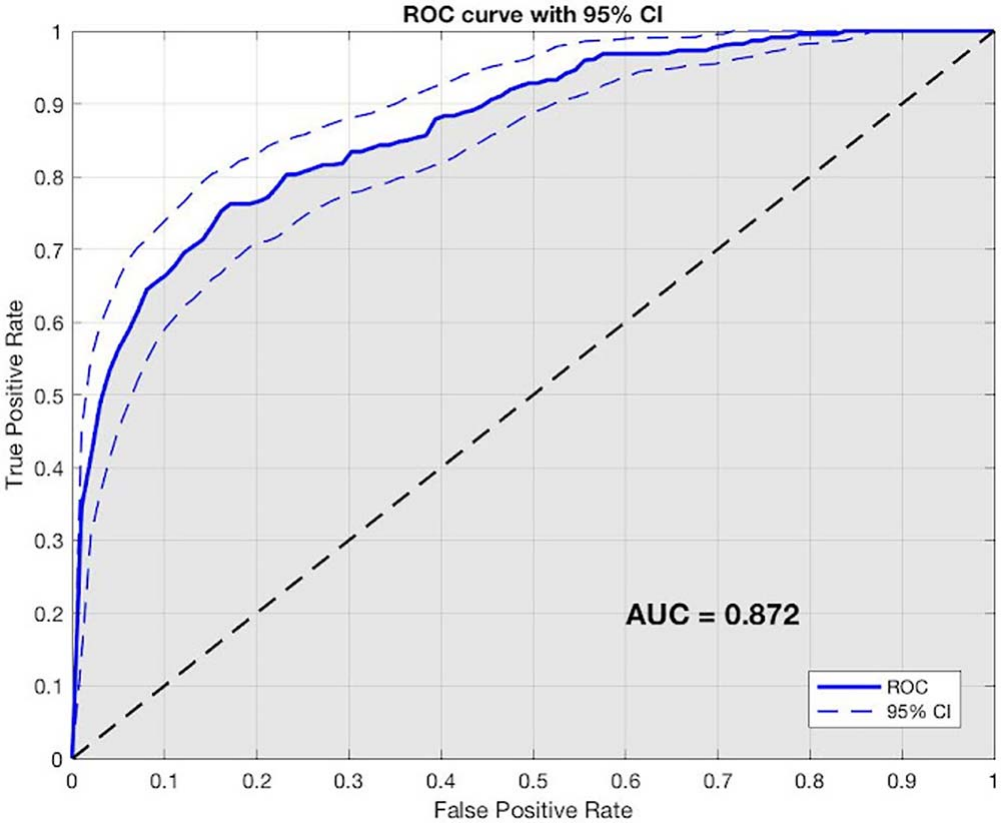
The ROC curve and AUC value with 95% CI derived from the logistic regression model. Abbreviations: AUC = area under the curve; ROC = receiver operating characteristic.

When these significant variables were included in the multivariate analysis, it was found that age (OR = 1.004, *P* < .001), NIHSS at the admission (OR = 1.032, *P* < .001), pre-stroke mRS (OR = 1.111, *P* < .001), hemorrhagic stroke (OR = 1.120, *P* < .001), sum of lobes (OR = 1.070, *P* < .001) and IEDs (OR = 1.088, *P* = .037) were independent predictors of poor outcome in terms of mRS 3-6 at 3 months (Table 2). The predictive logistic regression model, based on features identified as independent predictors in the multivariate analysis, achieved an accuracy of 81%. The receiver operating characteristic (ROC) curve and AUC value (0.872) with 95% CI derived from the model are shown in Figure 4 and the precision-recall curve (PRC) curve with AUPRC value (0.458) in the Supplementary material.

**Table 2:** Logistic multivariate regression for prediction of poor outcome at 3 months (mRS 3-6).

Variables	Odds ratio	95% CI	*P*-value
Age	1.004	1.002-1.007	**<.001**
NIHSS at admission	1.032	1.026-1.037	**<.001**
Pre-stroke mRS	1.111	1.074-1.148	**<.001**
Hemorrhagic stroke	1.210	1.106-1.323	**<.001**
Sum of lobes	1.070	1.039-1.102	**<.001**
IEDs	1.088	1.005-1.177	**.037**

Multivariate analysis for poor outcome at 3 months (mRS 3-6). Bold values for *P* < .05.

Abbreviations: CI = confidence interval; IEDs = interictal epileptiform discharges; mRS = modified Rankin scale; NIHSS = National Institutes of Health Stroke Scale.

## Discussion

The main finding of this study is that early IEDs on EEG within the first 72 h independently predict poor clinical outcomes at 3 months (mRS 3-6), along with age, NIHSS on admission, pre-stroke mRS, hemorrhagic stroke and lesion extension. The logistic regression model, based on these significant features including the presence on IEDs achieved a high accuracy of 81% in predicting functional outcomes. These results, obtained from a large study population of 593 acute stroke patients without symptomatic seizures, highlight the role of early EEG IEDs detection in predicting post-stroke clinical and functional outcomes.

In this context, EEG epileptiform abnormalities and seizures have been studied in stroke patients, with studies showing varying prevalence rates. The prevalence of acute symptomatic seizures in patients with cerebrovascular events ranges from 4% to 10%.^22–25^ Interictal epileptiform abnormalities have been observed in 25.7% of cases.^20^ In our sample, 18.2% of stroke patients exhibited EEG-detected IEDs within 72 h of admission. In particular, IEDs were observed in 91 of 516 patients with ischemic stroke (17.6%) and in 17 of 77 patients with hemorrhagic stroke (22.1%), confirming the higher prevalence of epileptiform alterations in the latter group, as previously reported in the literature.^20^ This increased incidence may be explained by the mass effect, cerebral edema and the pro-epileptogenic action of blood degradation products.^33^ These results confirm the significant prevalence of this neurophysiological alteration and the importance of EEG assessment.

Electroencephalography can be particularly valuable in acute stroke assessment and outcome prediction, considering both slowing and epileptiform patterns, especially in the hyper-acute phase. Specifically, EEG quantitative spectral features extracted in the early pre-treatment phase may contribute as objective parameters to the short/long-term outcome prediction.^14^ Moreover, quantitative EEG was also employed in the subacute stroke, confirming the EEG can contribute to the prognosis of stroke functional outcome and post-stroke attentional capacity.^16–18^ Furthermore, EEG was also correlated to neuroimaging alterations. Stragapede et al.^3^ and Ajčević et al.^2^ showed the correlation between EEG and perfusion alterations in hyper-acute stroke as a manifestation of neurovascular coupling. In particular, hemispheric prevalence of slow EEG rhythms and areas of cerebral hypoperfusion identified by CTP was observed^3^ and the correlation of hypoperfused volume and ischemic core volumes with EEG power band alterations were documented.^2^ Moreover, EEG and multimodal computed tomography, including CT perfusion, provide valuable information in emergency settings, particularly in differentiating ischemic stroke from seizure mimics in patients presenting with isolated aphasia.^6^

The stroke prognosis is still challenging; thus, new independent predictors are needed. The acute symptomatic seizures are independent predictors of mortality, while remote symptomatic seizures influence functional outcomes in the first year after stroke.^18^ However, to date, only one study, which also included patients with electrographic seizures, has investigated the relationship between clinical outcomes and EEG alterations including among them also epileptiform abnormalities.^26^ In particular, in a cohort of 131 patients 2 independent prognostic factors were identified for achieving a good functional status on the day of discharge: the neurological status as measured by the NIHSS on the first day and the absence of changes in the EEG in the contralateral unaffected hemisphere. In our study, involving a sample of 593 stroke patients who underwent EEG within 72 h of admission, the presence of IEDs was observed as an independent predictor of poor outcomes. Furthermore, patients whose EEGs displayed IEDs showed a higher prevalence of unfavourable mRS scores and a greater mortality rate at 3 months compared to those without IEDs. The NIHSS distribution at discharge among patients with IEDs also demonstrated greater variability and a higher proportion of cases with severe neurological deficit. These findings further support the importance of EEG assessment in the acute phase and suggest the potential value of this additional predictive EEG feature.

Interictal epileptiform discharges may arise as a consequence of acute ischemic brain injury, notably in its turn it can be a factor that exacerbates further damage. Hypermetabolism, hyperperfusion levels in the neural tissue has already been observed in patients with focal epileptic seizures and IEDs.^6^ The interplay between diminished metabolic capacity in the injured brain and the heightened metabolic demands associated with epileptiform activity may contribute to secondary neuronal injury.^34^^,^^35^ The post-ischemic inflammation occurs rapidly after the cerebrovascular event and is characterised by microglial activation, influx of peripheral immune cells and breakdown of the blood–brain barrier.^36^ In epilepsy research, chronic inflammation has also been delineated as a potent driver of epileptogenesis.^37^ Thus, this common element could also play an important role in the genesis of EEG alterations in the acute phase.

**Figure 5:**
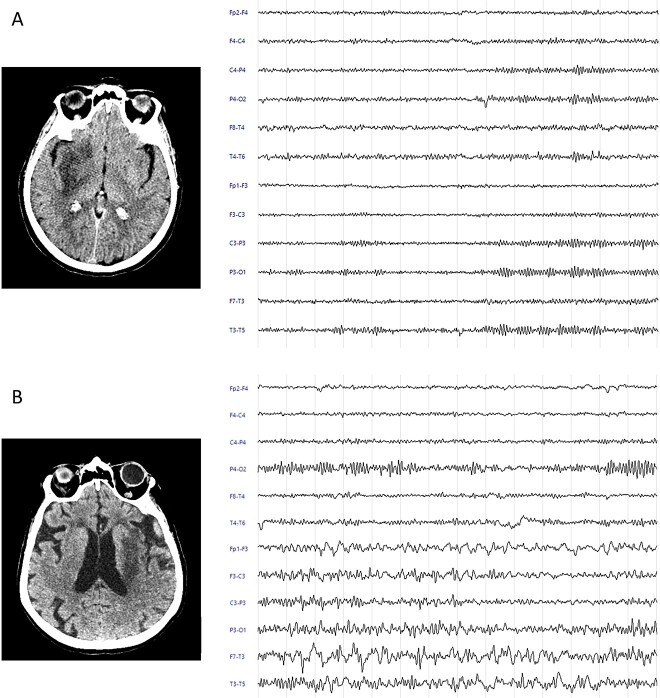
Representative electroencephalography (EEG) cases illustrating outcome-related patterns. (A) A 60-year-old woman with a history of hypertension presented to the emergency department 2 h after the onset of a moderate left-sided hemiparesis and dysarthria, with a National Institutes of Health Stroke Scale (NIHSS) score of 7. Initial non-contrast-enhanced computed tomography (NECT) imaging revealed no acute lesions (ASPECTS 10). Computed tomography angiography (CTA) demonstrated occlusion of the M1 segment of the right middle cerebral artery. The patient underwent reperfusion therapy with endovascular thrombectomy (EVT) and intravenous rt-PA. Follow-up NECT at 24 h showed a recent ischemic lesion in the fronto-insular lobes. A point-of-care EEG performed within the first 48 h showed no interictal epileptiform discharges (IEDs). The patient was discharged home with an NIHSS of 2 and a modified Rankin Scale (mRS) score of 2 at 3 months. (B) A 78-year-old woman with a history of atrial fibrillation and hypertension presented to the emergency department approximately 2 h after the onset of aphasia and right-sided motor hemiparesis (NIHSS 21). Non-contrast-enhanced CT (NECT) revealed a blurred loss of cortico-subcortical differentiation in the left temporal lobe (ASPECTS 9). Computed tomography angiography showed occlusion of the M1 segment of the middle cerebral artery. A follow-up NECT demonstrated hypodensities in the fronto-temporo-insular region, extending to the external capsule and lenticular nucleus on the left, consistent with an ischemic vascular lesion currently in the subacute phase. Point-of-care EEG performed within the first 48 h revealed frequent IEDs. The patient was discharged to a rehabilitation unit with an NIHSS of 14 and an mRS score of 4 at 3 months.

Other clinical features that emerged as significant predictors of poor outcome included advanced age, greater neurological deficit at admission as measured by the NIHSS, lesion extension, higher pre-stroke disability according to the mRS and hemorrhagic stroke. These factors have already been widely reported as predictors of poor outcome.^29^^,^^38^^,^^39^  Figure 5 illustrates 2 representative cases highlighting the distinguishing feature patterns linked to favourable and unfavourable outcomes.

## Limitations and future perspectives

This study has several limitations. First, it is a retrospective analysis, which may be subject to the influence of unmeasured confounding factors. Second, data were collected from a single Stroke Unit, which may limit the generalisability of the findings. Third, EEG characteristics were based on visual interpretation, relying on the expertise of trained neurophysiologists, which introduces the potential for inter-rater variability. Fourth, the prevalence of epileptiform abnormalities in the pre-stroke population remains unknown, and a point-of-care EEG approach was used rather than continuous EEG monitoring, potentially reducing the sensitivity for detecting transient epileptiform abnormalities. Fifth, no quantitative analysis of the EEG tracings was performed; therefore, alterations in spectral maps and slow-wave activity could not be incorporated into the outcome prediction model. Another limitation of this study is the class imbalance between the poor and good outcome groups, which may have influenced the model’s performance, as evidenced by the precision-recall curve, particularly in identifying the less frequent poor outcome cases. Finally, due to the heterogeneous distribution of IEDs severity and frequency, it was not possible to determine whether these factors would have enhanced the predictive accuracy for clinical outcomes.

Despite these limitations, this study enhances the understanding of prognostic factors associated with 3-month outcomes, as measured by the mRS, in a large patient cohort, and underscores the potential value of EEG in the acute phase of stroke. Future research should aim to identify the factors underlying the onset of IEDs in the acute phase, ideally using standardised EEG protocols, to determine whether the frequency and severity of epileptiform alterations enhance the predictive accuracy of clinical outcomes, and to assess whether their association with spectral map and slow-wave activity alterations can further refine predictive models of outcome. Furthermore, multicenter studies are needed to collect larger datasets of patients with hemorrhagic stroke to better clarify the potential role of IEDs in this condition.

## Conclusion

This study demonstrated that early IEDs, as assessed by point-of-care EEG within the first 72 h, are independent predictors of poor clinical outcomes at 3 months in stroke patients (mRS score 3-6), alongside age, NIHSS at admission, pre-stroke mRS, hemorrhagic stroke and lesion extent. Among the 593 stroke patients included in the study, 18.2% exhibited EEG-detected IEDs within 72 h of admission, confirming the notable prevalence of this neurophysiological finding and emphasising the clinical relevance of early EEG assessment. The developed logistic regression model achieved a clinically relevant accuracy in forecasting functional outcomes, and confirmed once again the importance of the presence of IEDs. These findings underscore the importance of EEG monitoring in the acute phase of stroke and suggest that early detection of IEDs may serve as a valuable additional prognostic marker.

## Supplementary Material

aakaf001_Supplementary_file

aakaf001_Ricci_25-0634VA

## Data Availability

The data will only be made available from the corresponding author upon reasonable request.
