# Supplementary material for: Interictal epileptiform activity in the acute stroke phase: an independent predictor of poor outcome
Source: Eur Stroke J. 2026 Jan 1;11(1):aakaf001. doi: 10.1093/esj/aakaf001 (PMC12964111; doi:10.1093/esj/aakaf001)

**Supplemental material**. The PRC curve and AUPRC value derived from the logistic regression model.


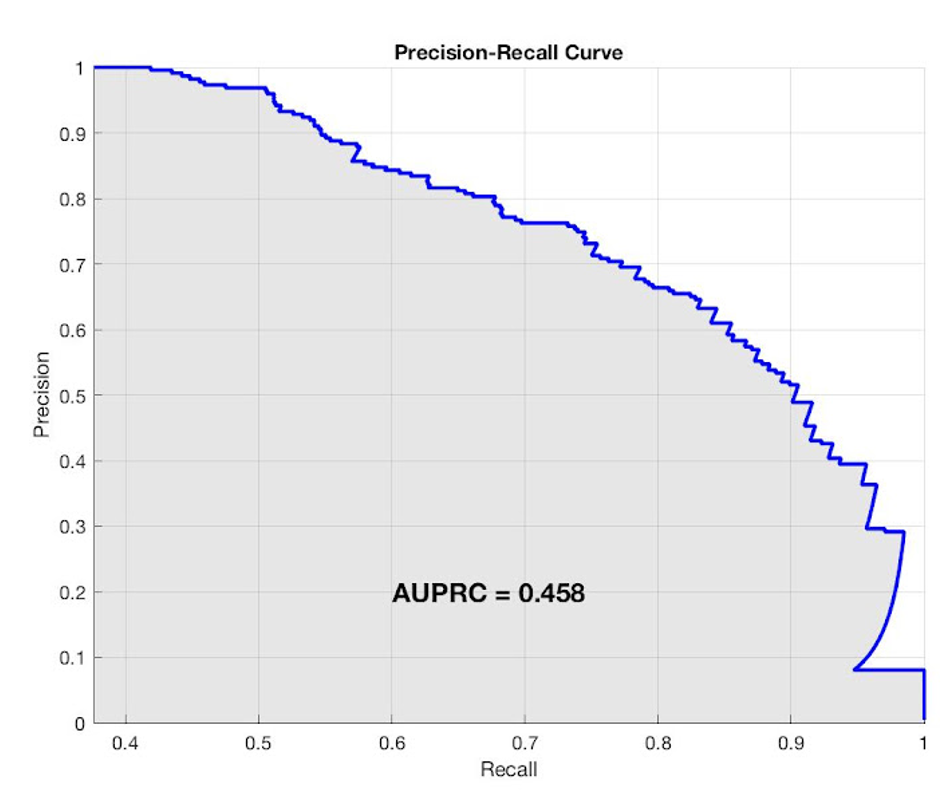

Supplement: aakaf001_Supplementary_file [file aakaf001_supplementary_file.docx]
