# Supplementary material for: Interictal epileptiform activity in the acute stroke phase: an independent predictor of poor outcome
Source: Eur Stroke J. 2026 Jan 1;11(1):aakaf001. doi: 10.1093/esj/aakaf001 (PMC12964111; doi:10.1093/esj/aakaf001)
Supplement: aakaf001_Ricci_25-0634VA [file aakaf001_ricci_25-0634va.pdf]

EUROPEAN STROKE JOURNAL

Interictal epileptiform activity in the acute stroke phase: an independent predictor of poor outcome

Are interictal epileptiform discharges (IEDs) within the first 72h of acute stroke predictive of functional outcome?

Methods

- Observational case-control study (2020–2023)
- 593 patients with acute stroke
- EEG within 72 h from admission
- Multivariate analysis for outcome prediction
- mRS: 0–2 vs. 3–6

Results

Early IEDs as independent predictor of poor outcome (mRS 3–6) at 3 months [OR: 1.088 (1.005–1.177)]

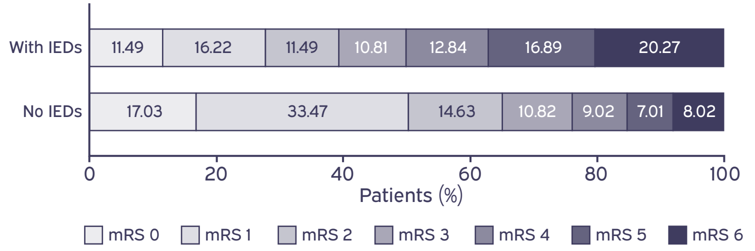

	No IEDs	With IEDs
mRS 3–6	34.87%	60.80%
Mortality	8.02%	20.27%

Conclusion

EEG-detected IEDs in the first 72h of acute stroke were independent predictors of poor functional outcome at 3 months.

Furlanis, G., et al. European Stroke Journal, 2025 edoardo.ricci@asugi.sanita.fvg.it doi.org/xx.xxxx/xxxxxxxxxxxxxxx
